# Diagnostic Criteria for Primary Autoimmune Cerebellar Ataxia—Guidelines from an International Task Force on Immune-Mediated Cerebellar Ataxias

**DOI:** 10.1007/s12311-020-01132-8

**Published:** 2020-04-23

**Authors:** Marios Hadjivassiliou, Francesc Graus, Jerome Honnorat, Sven Jarius, Maarten Titulaer, Mario Manto, Nigel Hoggard, Ptolemaios Sarrigiannis, Hiroshi Mitoma

**Affiliations:** 1 Academic Department of Neurosciences, Sheffield Teaching hospitals NHS Trust and University of Sheffield, Sheffield, UK; 2 Neuroimmunology Program, Institut d’Investigacions Biomediques August Pi Sunyer (IDIBAPS), Barcelona, Spain; 3 Hopital Neurologique, Lyon, France; 4 Molecular Neuroimmunology Group, Department of Neurology, University of Heidelberg, Heidelberg, Germany; 5 Department of Neurology, Erasmus MC University Medical Centre, Rotterdam, Netherlands; 6 Unite des Ataxies Cerebelleuses, CHU-Charleroi, Service des Neurosciences, UMons, Charleroi, Belgium; 7 Academic Department of Neuroradiology, Sheffield Teaching hospitals NHS Trust and University of Sheffield, Sheffield, UK; 8 Tokyo Medical University, Tokyo, Japan

**Keywords:** Primary autoimmune cerebellar ataxia (PACA), Immune ataxias

## Abstract

Aside from well-characterized immune-mediated ataxias with a clear trigger and/or association with specific neuronal antibodies, a large number of idiopathic ataxias are suspected to be immune mediated but remain undiagnosed due to lack of diagnostic biomarkers. Primary autoimmune cerebellar ataxia (PACA) is the term used to describe this later group. An International Task Force comprising experts in the field of immune ataxias was commissioned by the Society for Research on the Cerebellum and Ataxias (SRCA) in order to devise diagnostic criteria aiming to improve the diagnosis of PACA. The proposed diagnostic criteria for PACA are based on clinical (mode of onset, pattern of cerebellar involvement, presence of other autoimmune diseases), imaging findings (MRI and if available MR spectroscopy showing preferential, but not exclusive involvement of vermis) and laboratory investigations (CSF pleocytosis and/or CSF-restricted IgG oligoclonal bands) parameters. The aim is to enable clinicians to consider PACA when encountering a patient with progressive ataxia and no other diagnosis given that such consideration might have important therapeutic implications.

## Introduction

Immune-mediated cerebellar ataxias (IMCA) include ataxias where the trigger is known, e.g. paraneoplastic cerebellar degeneration (PCD) [1], gluten ataxia (GA) [2], post-infectious cerebellitis (PIC) [3] as well as ataxias where neuronal antibodies have been convincingly shown to be directly involved in the pathogenesis of the ataxia. The term primary autoimmune cerebellar ataxia (PACA) was introduced to describe a group of patients with suspected IMCA in which neither a trigger nor any pathogenic neuronal antibodies have been discovered as yet [4].

A task force comprising clinicians with an interest and extensive clinical experience in the management of IMCA was formed in 2017 at the request of the Society for Research on the Cerebellum and Ataxias (SRCA). The aim of this international task force was to use their clinical expertise in devising consensus diagnostic criteria in an attempt to assist clinicians to suspect PACA as a potential diagnosis amongst patients with otherwise idiopathic sporadic ataxia. This is a very critical step enabling the consideration of early therapy aiming to preserve or restore cerebellar reserve.

## Primary Autoimmune Cerebellar Ataxia (PACA)

**Fig. 1:**
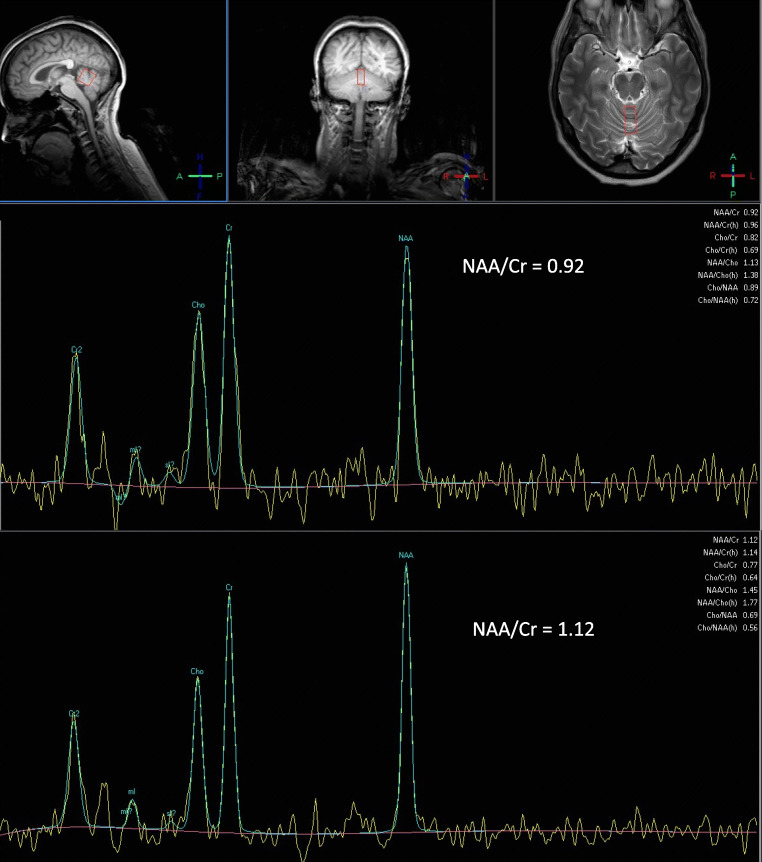
MR spectroscopy of the cerebellar vermis of a patient with PACA before and a year after treatment with mycophenolate. Note the improvement of the NAA/Cr area ratio from 0.92 pre-treatment to 1.12 after treatment. This was associated with clinical improvement

The task force proposes that the term primary autoimmune cerebellar ataxia (PACA) should encompass all ataxias that fulfil the criteria outlined in Fig. 1 with the following clarifications: PACA can be associated with neuronal antibodies. However, the term PACA should not be used if such neuronal antibodies have already been shown to be directly involved in the pathogenesis of the ataxia (e.g. DPPX, mGluR1, GABABR, anti-GAD) or are markers of ataxias with a known trigger (e.g. anti-Yo in PCD or antigliadin antibodies in GA). The task force recognizes the possibility of future clarification of antibody pathogenicity in patients that currently meet the criteria for PACA. In the event of such discovery, the ataxia in question would no longer come under the umbrella of PACA but would bear the name of the specific pathogenic antibody (e.g. DPPX ataxia). The task force acknowledges that ataxias are designated as PACA (as per criteria below) although all immune mediated will ultimately prove heterogeneous, in terms of both pathogenesis and optimum treatment. However, the consideration and recognition of PACA should alert the clinician to the possibility of a potentially treatable ataxia which is the primary aim of this work.

## Clinical Pointers to PACA

Most immune-mediated ataxias have a predilection for vermian involvement although hemispheric involvement is not common [5]. The clinical correlate is that of gait ataxia either in isolation or more prominent than limb ataxia. This is in contrast to most genetic and degenerative ataxias where the cerebellar involvement is usually more global affecting both vermis and hemispheres equally, resulting in both limb incoordination, speech involvement as well as gait instability.

Genetic ataxias tend to progress slowly (usually over many years) with often a poorly defined temporal onset uncommon. Most IMCAs are of acute (days) or subacute (weeks or months) onset. Progression as a rule is faster in immune than in genetic ataxias, and immune ataxias follow a progressive course when untreated. The only non-immune-mediated ataxia where progression can be fast is cerebellar variant of multiple system atrophy (MSA-C). However a number of additional features should allow the distinction of MSA-C from PACA. In MSA-C autonomic dysfunction (urinary and postural symptoms, impotence, vivid dreams, sleep apnoea), early speech involvement, global ataxia, sometimes extrapyramidal features and characteristic MR imaging appearances (usually later on in the disease process) with atrophy of the pons and “hot cross bun” sign and selective hypometabolism on 18-FDG PET are all common features [6]. Careful identification of these features helps the differential diagnosis of MSA-C from IMCA.

It is extremely rare for an IMCA to be associated with a family history of ataxia. Therefore, the presence of a family history of ataxia suggests a genetic rather than an immune ataxia. It is not uncommon, however, for either the patient with immune ataxia or their first-degree relative being affected by another autoimmune disease [7]. Indeed this observation should alert to the possibility of PACA. Common examples of such autoimmune diseases include thyroid autoimmunity, Sjogren’s syndrome, pernicious anaemia, type 1 diabetes mellitus (DM) and vitiligo.

## Investigations Pointing to PACA

The presence of antibodies that suggest autoimmune tendency can be helpful in the diagnosis of IMCA. Such antibodies are not always pathogenic, may not necessarily be anti-neuronal but can simply signify tendency to autoimmunity even in the absence of a concurrent autoimmune disease (e.g. presence of thyroid peroxidase, intrinsic factor antibodies, anti-Ro). There is an ever-increasing number of anti-neuronal autoantibodies that have been identified in the context of immune-mediated neurological diseases, including ataxias [8] (see Table 1). Such anti-neuronal autoantibody tests are often not readily available even in regional immunology laboratories. In addition these antibodies are not necessarily pathogenic or well-characterized but still may provide some indirect evidence for PACA.

**Table 1:** Some examples of autoantibodies observed in primary autoimmune cerebellar ataxia

1 Examples of autoantibodies associated with non-neurological autoimmune diseases that may raise suspicion of PACA	
Thyroid peroxidase, thyroglobulin	PACA, thyroid autoimmune diseases
Anti-SS_A_ (Ro), SS_B_ (La)	PACA, Sjogren’s syndrome
2 Examples of autoantibodies reported only in a few patients with ataxia, thus significance in the context of ataxia is less well characterized but their presence may raise suspicion of PACA	
*Autoantibody*	*Clinical features*
Anti-Sj/ITPR-1	Variable clinical course
Anti-Ca/ARHGAP26	Variable clinical course. Association with neoplasm reported
Anti-MAG	Chronic gait ataxia and neuropathy. Ataxia is central in origin
Anti-Septin-5	Chronic cerebellar syndrome, no improvement after immunotherapy
Anti-neurochondrin	Chronic cerebellar/brainstem syndrome
Anti-Nb/AP3B2	Subacute cerebellar ataxia
Anti-Homer-3	Subacute cerebellar ataxia

ITPR1, inositol 1,4,5-triphosphate receptor type 1; ARHGAP26, Rho GTPase-activating protein 26; MAG, myelin-associated glycoprotein

Cerebrospinal fluid examination can sometimes provide some pointers to an immune pathogenesis with the presence of pleocytosis (anything above normal range for white cells) and/or CSF-restricted IgG oligoclonal bands [9, 10].

MR imaging of the cerebellum may also provide further support with evidence of preferential involvement of the vermis (at least at the initial presentation) in the context of PACA. In addition, if available MR spectroscopy of the vermis (measuring N-acetyl aspartate to creatine, NAA/Cr ratio) can be a very sensitive test of cerebellar dysfunction and a useful monitoring tool particularly when considering immunosuppression (Fig. 1) [11, 12].

There is some emerging evidence to suggest that immune-mediated ataxias are more likely to be linked to “brain hyperexcitability” (e.g. DPPX ataxia, myoclonic ataxia due to refractory coeliac disease) as indicated by cortical myoclonus (using back averaging), giant somatosensory evoked potentials, abnormal blink reflex and exaggerated startle responses [13, 14].

Immunohistochemistry using sera or CSF from patients with suspected PACA often shows reactivity with cerebellar tissue, which is not the case when using sera from patients with genetic ataxias [7].

Accessibility to tissue for biopsy, such as, for example, peripheral nerve in the context of any concurrent peripheral neuropathy or skin in the presence of a vasculitic skin rash, may also be helpful in alerting to the possibility of PACA.

Given that PACA, as proposed in this article, is a new entity and that it is likely to prove to be heterogeneous, it is difficult to put emphasis on any PM pathological findings based on what is available in the literature. Furthermore, most of the literature refers to well-characterized immune-mediated ataxias such as PCD and GA [15, 16].

## Diagnostic Criteria for PACA (Fig. 2)

The following criteria need to be fulfilled to be able to diagnose PACA:

1. Predominantly subacute or acute pure cerebellar syndrome (gait ataxia that may be associated with variable degrees of limb incoordination, dysarthria, nystagmus, diplopia)
2. MRI at presentation usually normal or may show primarily cerebellar vermian atrophy with (if available) reduced MR spectroscopy (NAA/Cr ratio) of the vermis
3. At least 2 of the following:
  - CSF pleocytosis and/or positive CSF-restricted IgG oligoclonal bands
  - History of other autoimmune disorders or family history of autoimmune disorders in first-degree relatives
  - Presence of antibodies that support autoimmunity but not yet shown to be either directly involved in ataxia pathogenesis (Table 1) or to be markers of ataxia with a known trigger
4. Exclusion of alternative causes made by an experienced neurologist or ataxia specialist (including other causes of immune ataxia such as PCD, GA, PIC and ones that are associated with well-characterized pathogenic antibodies)

**Fig. 2:**
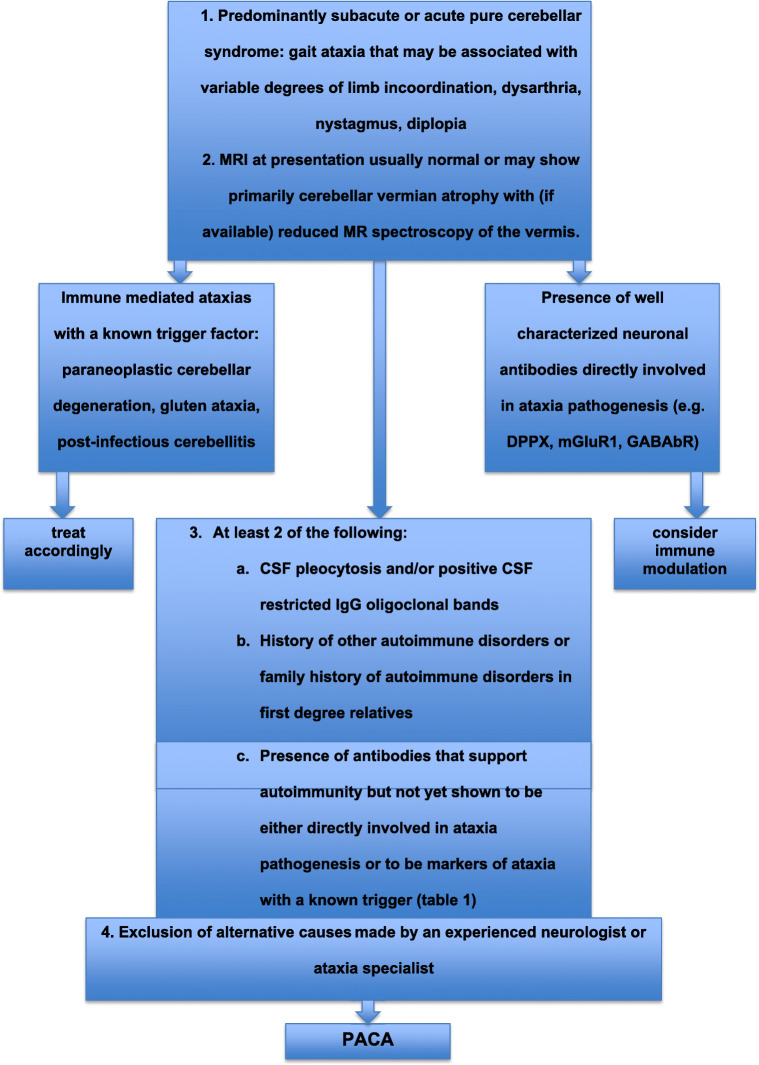
Flow diagram showing the diagnostic criteria for PACA in the context of other immune-mediated ataxias

## Discussion

This is the first attempt to devise consensus diagnostic criteria for PACA by an international task force comprising experts in the field of immune ataxias. The task was commissioned by the Society for Research on the Cerebellum and Ataxias (SRCA). The significant progress in the genetic characterization of the ataxias has highlighted the fact that in a large number of patients with idiopathic late onset ataxia and no family history of ataxia (sporadic), the ataxia is unlikely to be due to any known genetic defect or due to neurodegeneration. A potential autoimmune pathogenesis should be considered for such patients.

In a recent prospective study, out of a total of 1500 patients with progressive ataxia, IMCAs accounted for 26% [17]. In the same series, idiopathic sporadic ataxias accounted for 20% of all ataxias. It is amongst these 20% that PACA can be potentially identified and hopefully diagnosed using the proposed criteria.

It has been argued that a substantial number of this 20% of patients with idiopathic sporadic ataxia still have a genetic defect that accounts for their ataxia but has not as yet been identified. Despite the rapid development in genetic testing, including new generation sequencing, screening of 114 patients with sporadic idiopathic adult onset ataxia, attending the Sheffield Ataxia Centre, using a panel containing 175 genes associated with ataxia, identified a genetic cause in just 6% (personal observation).

Degenerative causes of ataxia with a well-established pathological characterization currently only include MSA-C, an entity that is clinically very distinct. There is no evidence of a common neurodegenerative pathological mechanism that would potentially characterize or account for some of the 20% of idiopathic sporadic ataxias. Within this group, it is likely that a substantial number of patients have PACA. Correctly diagnosing patients with PACA is of potential therapeutic importance.

A retrospective study showed that amongst 118 patients with suspected IMCA, 55 had non-paraneoplastic ataxia [18]. The majority of these 55 patients had ataxia associated with well-characterized neuronal antibodies (i.e. not PACA). All 118 patients had received some form of immunotherapy, and neurological improvement was reported in 54 patients (46%). Regression analysis revealed that improvements were significantly more common amongst patients with non-paraneoplastic ataxias.

In conclusion, we present here for the first time the consensus diagnostic criteria in an attempt to aid the diagnosis of PACA. Our aim is to facilitate the diagnosis of PACA in order to instigate appropriate immunotherapy, gather a large number of cases into a registry and allow clinical trials. These criteria will help elucidate the epidemiology and pathogenesis of PACA. As with any criteria, they are subject to future improvements in order to refine and improve diagnosis.
